# Supplementary material for: Machine Learning Accelerates Crystallization for Structure Determination
Source: Angew Chem Int Ed Engl. 2026 May 4;65(25):e1218503. doi: 10.1002/anie.1218503 (PMC13266958; doi:10.1002/anie.1218503)

checkCIF/PLATON report

Structure factors have been supplied for datablock(s) lcz038

THIS REPORT IS FOR GUIDANCE ONLY. IF USED AS PART OF A REVIEW PROCEDURE FOR PUBLICATION, IT SHOULD NOT REPLACE THE EXPERTISE OF AN EXPERIENCED CRYSTALLOGRAPHIC REFEREE.

No syntax errors found. CIF dictionary Interpreting this report

Datablock: lcz038

Bond precision:	C-C = 0.0122 A		Wavelength=1.54184
Cell:	a=13.5121(2)	b=12.8040(2)	c=20.7098(3)
	alpha=90	beta=99.861(1)	gamma=90
Temperature:	100 K		

	Calculated	Reported
Volume	3530.05(9)	3530.05(9)
Space group	P 21/n	P 1 21/n 1
Hall group	-P 2yn	-P 2yn
Moiety formula	C15 H3 Ag3 F18 N6, C15 H12 O2	C15 H3 Ag3 F18 N6, C15 H12 O2
Sum formula	C30 H15 Ag3 F18 N6 O2	C30 H15 Ag3 F18 N6 O2
Mr	1157.09	1157.09
Dx,g cm-3	2.177	2.177
Z	4	4
Mu (mm-1)	14.495	14.495
F000	2224.0	2224.0
F000’	2234.55	
h,k,lmax	17,16,26	17,16,26
Nref	7616	7285
Tmin,Tmax	0.293,0.235	0.167,1.000
Tmin’	0.188	

Correction method= # Reported T Limits: Tmin=0.167 Tmax=1.000
AbsCorr = MULTI-SCAN

```
Data completeness= 0.957          Theta(max)= 78.868

R(reflections)= 0.0587( 6051)                      wR2(reflections)=
                                                   0.1512( 7285)
S = 1.071                 Npar= 533
```

The following ALERTS were generated. Each ALERT has the format
test-name_ALERT_alert-type_alert-level.
Click on the hyperlinks for more details of the test.

Alert level C

```
PLAT213_ALERT_2_C Atom F00O           has ADP max/min Ratio .....        3.1 prolat
PLAT213_ALERT_2_C Atom F00S           has ADP max/min Ratio .....        3.5 prolat
PLAT213_ALERT_2_C Atom F00Y           has ADP max/min Ratio .....        3.5 prolat
PLAT342_ALERT_3_C Low Bond Precision on  C-C Bonds ..............    0.01218 Ang.
PLAT906_ALERT_3_C Large K Value in the Analysis of Variance ......      7.874 Check
PLAT906_ALERT_3_C Large K Value in the Analysis of Variance ......      2.202 Check
PLAT911_ALERT_3_C Missing FCF Refl Between Thmin & STh/L=    0.600         30 Report
               10  0  0,   1  0  1,   0  0  2,  -9  0  7, -16  0  8,   9 10  9,
                9 10 10,  10  4 15,   8  3 16,   9  3 16,   9  4 16,   9  5 16,
               10  0 16,   5  1 17,   7  3 17,   8  2 17,   8  4 17,   9  0 17,
                3  7 18,   4  7 18,   8  0 18,   7  0 19,   8  1 19,   7  1 20,
                7  2 20,  -3  8 21,  -4  4 22,  -4  6 22,  -3  6 22,  -3  7 22,
```

Alert level G

```
PLAT003_ALERT_2_G Number of Uiso or U(i,j)  Restrained non-H-Atoms          2 Report
PLAT007_ALERT_5_G Number of Unrefined Donor-H Atoms .............          1 Report
              H00B
PLAT083_ALERT_2_G SHELXL Second Parameter in WGHT  Unusually Large      49.80 Why ?
PLAT186_ALERT_4_G The CIF-Embedded .res File Contains ISOR Records          1 Report
PLAT242_ALERT_2_G Low    'MainMol' Ueq as Compared to Neighbors of       C00Z Check
PLAT242_ALERT_2_G Low    'MainMol' Ueq as Compared to Neighbors of       C015 Check
PLAT242_ALERT_2_G Low    'MainMol' Ueq as Compared to Neighbors of       C017 Check
PLAT242_ALERT_2_G Low    'MainMol' Ueq as Compared to Neighbors of       C01D Check
PLAT242_ALERT_2_G Low    'MainMol' Ueq as Compared to Neighbors of       C01H Check
PLAT242_ALERT_2_G Low    'MainMol' Ueq as Compared to Neighbors of       C01J Check
PLAT720_ALERT_4_G Number of Unusual/Non-Standard Labels ..........         74 Note
              Ag01    Ag02    Ag03    F004    F005    F006    F007    F008
              F009    O00A    O00B    H00B    F00C    F00D    F00E    F00F
              F00G    N00H    N00I    N00J    F00K    N00L    N00M    N00N
              F00O    F00P    F00Q    F00R    F00S    C00T    H00T    C00U
              H00U    C00V    C00W    C00X    F00Y    C00Z    C010    C011
              C012    H012    C013    H013    C014    H014    C015    C016
              C017    C018    C019    C01A    C01B    H01B    C01C    H01C
              C01D    C01E    H01E    C01F    C01G    H01G    C01H    C01I
              H01I    C01J    C01K    H01K    C01L    H01L    C01M    H01M
              C01N    H01N
PLAT860_ALERT_3_G Number of Least-Squares Restraints ............         12 Note
PLAT883_ALERT_1_G Absent Datum for _atom_sites_solution_primary ..     Please Do !
PLAT912_ALERT_4_G Missing # of FCF Reflections Above STh/L=  0.600        301 Note
PLAT933_ALERT_2_G Number of HKL-OMIT Records in Embedded .res File         14 Note
               -4  6 23,   8  4 17,   5  1 17,   8  2 17,   9  5 16,   9  3 16,
```

```
              10   5 15,    7   3 17,   10   4 15,   -4   6 24,   11   5 14,    8   3 16,
               9   4 16,    9 10   9,
PLAT941_ALERT_3_G Average HKL Measurement Multiplicity ..........        2.7 Low
PLAT969_ALERT_5_G The 'Henn et al.' R-Factor-gap value ..........      3.370 Note
             Predicted wR2: Based on SigI**2  4.49 or SHELX Weight 14.12
PLAT978_ALERT_2_G Number C-C Bonds with Positive Residual Density.          0 Info
```

```
   0 ALERT level A = Most likely a serious problem - resolve or explain
   0 ALERT level B = A potentially serious problem, consider carefully
   7 ALERT level C = Check. Ensure it is not caused by an omission or oversight
  18 ALERT level G = General information/check it is not something unexpected

   1 ALERT type 1 CIF construction/syntax error, inconsistent or missing data
  13 ALERT type 2 Indicator that the structure model may be wrong or deficient
   6 ALERT type 3 Indicator that the structure quality may be low
   3 ALERT type 4 Improvement, methodology, query or suggestion
   2 ALERT type 5 Informative message, check
```

Validation response form

Please find below a validation response form (VRF) that can be filled in and pasted into your CIF.

```
# start Validation Reply Form
_vrf_PLAT213_lcz038
;
PROBLEM: Atom F00O            has ADP max/min Ratio .....        3.1 prolat
RESPONSE: ...
;
_vrf_PLAT342_lcz038
;
PROBLEM: Low Bond Precision on  C-C Bonds ..............    0.01218 Ang.
RESPONSE: ...
;
_vrf_PLAT906_lcz038
;
PROBLEM: Large K Value in the Analysis of Variance ......      7.874 Check
RESPONSE: ...
;
_vrf_PLAT911_lcz038
;
PROBLEM: Missing FCF Refl Between Thmin & STh/L=    0.600         30 Report
RESPONSE: ...
;
# end Validation Reply Form
```

It is advisable to attempt to resolve as many as possible of the alerts in all categories. Often the minor alerts point to easily fixed oversights, errors and omissions in your CIF or refinement strategy, so attention to these fine details can be worthwhile. It is up to the individual to critically assess their own results and, if necessary, seek expert advice.

PLATON version of 17/09/2025; check.def file version of 17/09/2025

duplicate check

No duplication found

Datablock lcz038 - ellipsoid plot

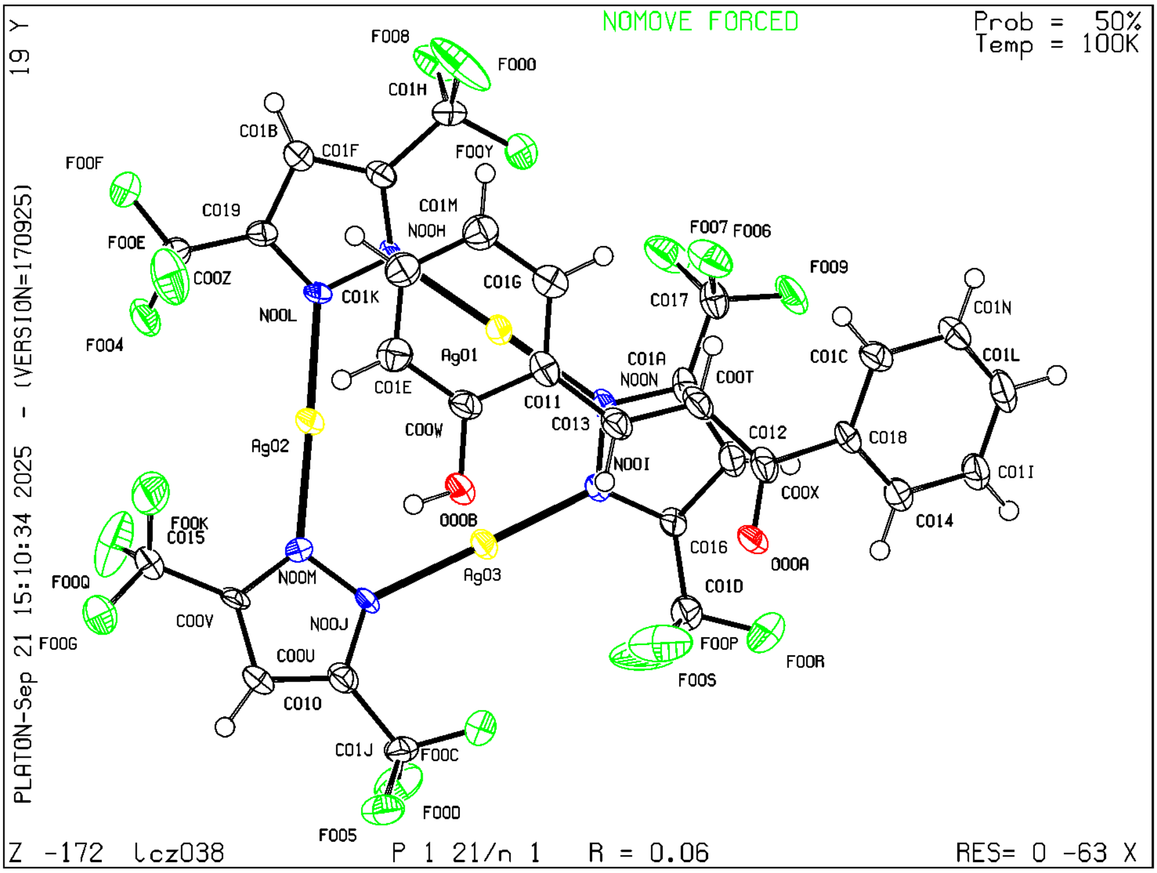

Supplement: Supplementary file 2 — Supporting File 2: anie72462‐sup‐0002‐DataFile.zip. [file ANIE-65-e1218503-s002.zip › supplementary_materials_crystallographic_data submit.zip!/cif_and_checkcif(1-114)/Ag3Pz3_38/Ag3Pz3_38_checkcif.pdf]
